# Generation of a reference transcriptome for evaluating rainbow trout responses to various stressors

**DOI:** 10.1186/1471-2164-12-626

**Published:** 2011-12-21

**Authors:** Cecilia C Sánchez, Gregory M Weber, Guangtu Gao, Beth M Cleveland, Jianbo Yao, Caird E Rexroad

**Affiliations:** 1 Shepherd University, Institute of Environmental and Physical Sciences, Robert C. Byrd Science and Technology Center, Shepherdstown, WV, 25443, USA; 2 USDA/ARS/NCCCWA, 11861 Leetown, Kearneysville, WV, 25430, USA; 3 West Virginia University, Animal and Nutritional Sciences, Morgantown, WV, 26506, USA

## Abstract

**Background:**

Fish under intensive culture conditions are exposed to a variety of acute and chronic stressors, including high rearing densities, sub-optimal water quality, and severe thermal fluctuations. Such stressors are inherent in aquaculture production and can induce physiological responses with adverse effects on traits important to producers and consumers, including those associated with growth, nutrition, reproduction, immune response, and fillet quality. Understanding and monitoring the biological mechanisms underlying stress responses will facilitate alleviating their negative effects through selective breeding and changes in management practices, resulting in improved animal welfare and production efficiency.

**Results:**

Physiological responses to five treatments associated with stress were characterized by measuring plasma lysozyme activity, glucose, lactate, chloride, and cortisol concentrations, in addition to stress-associated transcripts by quantitative PCR. Results indicate that the fish had significant stressor-specific changes in their physiological conditions. Sequencing of a pooled normalized transcriptome library created from gill, brain, liver, spleen, kidney and muscle RNA of control and stressed fish produced 3,160,306 expressed sequence tags which were assembled and annotated. SNP discovery resulted in identification of ~58,000 putative single nucleotide polymorphisms including 24,479 which were predicted to fall within exons. Of these, 4907 were predicted to occupy the first position of a codon and 4110 the second, increasing the probability to impact amino acid sequence variation and potentially gene function.

**Conclusion:**

We have generated and characterized a reference transcriptome for rainbow trout that represents multiple tissues responding to multiple stressors common to aquaculture production environments. This resource compliments existing public transcriptome data and will facilitate approaches aiming to evaluate gene expression associated with stress in this species.

## Background

Fish under intensive culture conditions are exposed to a variety of acute and chronic stressors, including elevated rearing densities, sub-optimal water quality including decreased dissolved oxygen (DO) and high carbon dioxide (CO_2_), and thermal fluctuations [1,2]. During transportation and sorting, several factors may also lead to stress including handling and tank confinement [3,4]. Osmoregulatory disturbance may be an outcome of stress, for which altered salinity is routinely used to mitigate negative effects, however high salinity such as in adaptation to seawater induces a stress response [5]. These and other stressors which are common to aquaculture production can induce physiological responses which may have adverse effects on traits important to producers and consumers, including those associated with growth, nutrition, reproduction, immune response, fillet quality, and environmental impacts [1,2,6-13]. Understanding and monitoring the biological mechanisms underlying stress responses will facilitate alleviating their negative effects through selective breeding and changes in management practices, resulting in improved animal welfare and production efficiency.

Studies which aim to characterize global gene expression in response to stress often use hybridization-based approaches (i.e. microarrays) to identify differences between challenged and control fish [14-16]. Hybridization techniques including those that employ microarrays can be high-throughput and are relatively inexpensive, however they present some limitations [17]. For instance: 1) the preparation of custom-made arrays requires previous knowledge of the target transcriptome expected to be represented in a sample; 2) hybridization data often present high background levels due to cross-hybridization; and 3) microarrays are not suitable for genes expressed at very low or very high levels because there is a limited range of detection due to background and saturation [18]. Sequencing-based methodologies have also been used to characterize gene expression, full-length cDNA and EST (expressed sequence tags) libraries have been sequenced using Sanger technology, however, this method is low-throughput, expensive and usually not quantitative [17,18]. Tag-based short sequence reads methodologies like SAGE (serial analysis of gene expression) or MPSS (massive parallel signature sequence) are also high-throughput and quantitative, however the sequence reads cannot be unambiguously mapped to the reference genome; the transcriptome is still partially

analyzed; and it is usually not possible to distinguish different gene isoforms [18,19]. Recently developed high-throughput sequencing technologies offer a new approach (RNASeq) for characterizing transcriptomes through high-throughput sequencing, mapping and quantification [20-31]. This approach is especially advantageous for non-model species, because it is not restricted by the unavailability of a genome reference sequence. Additionally, RNASeq has very low background and sequences can be unambiguously mapped to reference sequences. The quantification is based on the number of sequences obtained, therefore it offers the detection of a higher expression range and it is possible to identify low or highly expressed genes. Furthermore, this approach permits additional analysis such as the identification of putative single nucleotide polymorphisms (SNPs), which can be identified in transcribed regions and have the potential to affect gene function [32]. RNASeq has previously been used for transcriptome characterization of non-model species, including butterfly [20], silkworm [21], garter snake [22], coral [23], pearl oyster [24] and several fish species [25-30], including rainbow trout [31].

In the absence of a genome reference sequence for rainbow trout, we used Roche 454 pyrosequencing technology to develop a reference transcriptome sequence to be used specifically in gene expression analyses associated with stress, including RNASeq based approaches. The library construction included a normalization process [22,23,33] such that the reference transcriptome would constitute a qualitative resource attempting to represent the maximum number of unique transcripts from each of the tissues/treatment combinations as possible [26]. Although the majority of stress gene expression research in rainbow trout has concentrated on evaluating the effect of only one stressor on one specific tissue [34-36], our goal was to capture and characterize a collection of transcripts from multiple tissues from fish exposed to one of five stressors. As a result we aim to establish a comprehensive stress transcriptome resource that will facilitate understanding stress responses in this species. To this end we selected stressors that are among those commonly experienced by rainbow trout during aquaculture production and transport including high temperature, low temperature, low DO/high CO_2_, seawater transfer, and handling/confinement.

Previous studies have evaluated the combined effects of temperature and salinity on physiological condition of rainbow trout. Niu *et al. *[37] transferred juvenile rainbow trout from 13.5°C to 25.5°C, held them for two hours, and then transferred them to a 32‰ water at 13.5°C to observe the effects osmotic stress during transfer from freshwater to saltwater. Also, low temperature combined with sea water tolerance was analyzed by Findstad *et al *[38] who transferred fresh water acclimated rainbow trout to sea water at 1 and 8°C, and Saunders *et al *[39] who first acclimated the fish to salt water and placed them in floating cages until temperatures fell below 0°C. In this project we used similar parameters for high and low temperature and salinity treatments however we were conducted treatments independently. Previous studies have suggested that oxygen concentrations above 6 mg/l are not limiting factors for growth [40] and carbon dioxide in culture tanks should range between 10 and 20 mg/l [41] therefore we combined effects of low DO and high CO_2 _by supplying fish with re-use water, a common practice in aquaculture production which uses water from rearing sites "upstream" to increase production capacity [42]. Finally, fish were subjected to a handling and confinement stress routinely used for rainbow trout stress response research [15,43-46].

We demonstrate that each stressor produced a physiological response by reporting changes in plasma variables indicative of various phases of a stress response (cortisol, glucose, lactate, and chloride concentrations and lysozyme activity [47-49]) as well as changes in the expression of genes in gill tissue related to apoptosis [50-52] and Na/K transport. Our reference transcriptome created by sequencing RNA from multiple tissues of control and stressed fish is inclusive of transcripts expressed within the context of these unique physiological states and typical basal expression from control fish. We characterize the reference transcriptome by conducting an assembly of sequencing reads, assigning Gene Ontology annotation [53] and identifying putative SNPs.

## Results and Discussion

### Water Quality Parameters

Water quality parameters including temperature, DO, pH, CO_2_, and ammonia were measured at 0, 3, and 6 hours during the challenges and as summarized in Additional File 1 (Table S1 Water Quality Parameters). The desired parameters for each treatment were obtained within three hours of the beginning of the respective experiments. Water pH values varied between 7.5 and 7.9 for both treatment and control conditions. Concentrations of nitrogenous compounds levels were also within acceptable limits. In the "high temperature" treatment tanks, temperatures were elevated from 14.3°C to an average of 22.6°C within three hours (time 3) and remained elevated until the six hour (time 6) sampling period, at which temperatures averaged 24.8°C. For the "low-temperature" treatment, water temperature was reduced to 1.8°C within three hours (time 3), and was maintained at an average of 2.3°C until time 6. DO levels are one of the most critical water quality parameters in trout culture and exposure to low levels of DO (5.0 - 6.0 mg/l) can result in mortality [54]. However, critical levels are directly correlated with other water quality parameters, like water temperature. DO levels oscillated between 7.16 and 22.40 mg/l across the experimental tanks. Although the average DO concentration at sampling time (time 6) was lower in the re-use water treatment tanks than the control tank (7.8 versus 10.0 mg/l), it remained within acceptable limits for trout production. However, CO_2 _levels were significantly higher (67.8 mg/l average) in treatment tanks compared to control (21.7 mg/l). Recommended upper limits for dissolved CO_2 _range between 10 and 20 mg/l [55].

### Physiological responses

**Figure 1:**
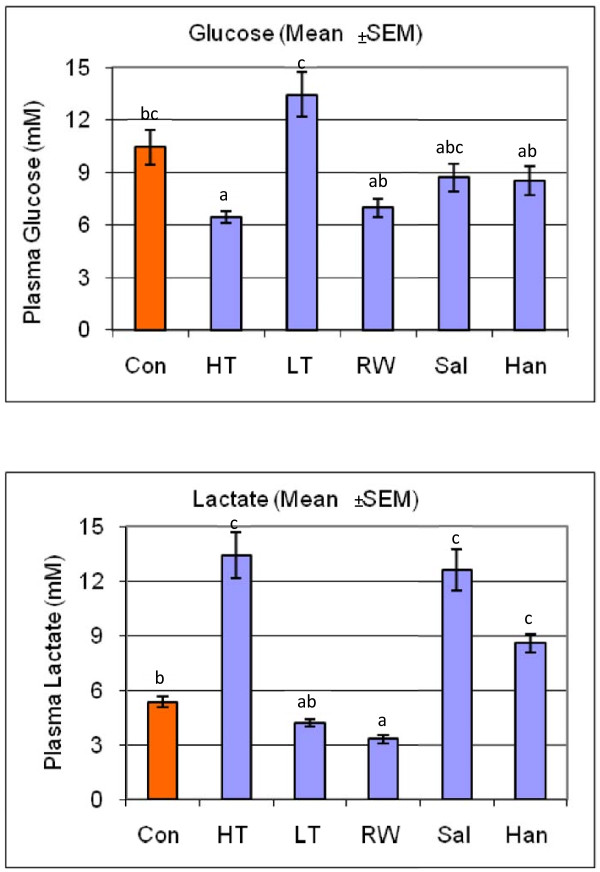
**Physiological Responses to Stress Treatments**. The response of plasma glucose and lactate across all treatments and controls. Fish treatments included Control (Con), High Temperature (HT), Low Temperature (LT), Re-use Water (RW), Salinity (Sal) and Handling/Confinement (Han). Values from all control tanks were pooled and presented as a single mean, which was compared to the mean for each stress treatment.

**Figure 2:**
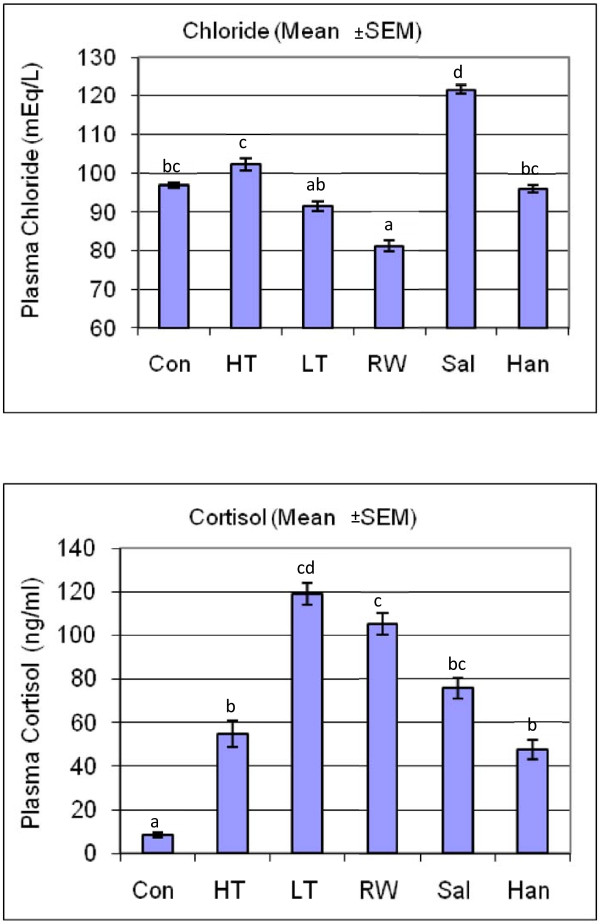
**Physiological Responses to Stress Treatments**. The response of plasma chloride and cortisol concentrations compared across all treatments and controls. Fish treatments included Control (Con), High Temperature (HT), Low Temperature (LT), Re-use Water (RW), Salinity (Sal) and Handling/Confinement (Han). Values from all control tanks were pooled and presented as a single mean, which was compared to the mean for each stress treatment.

**Figure 3:**
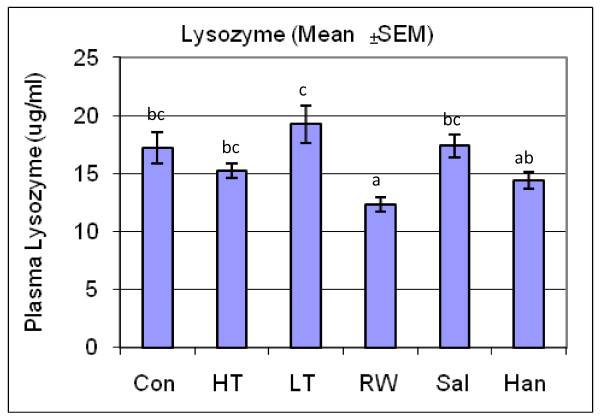
**Physiological Responses to Stress Treatments**. The response of lysozyme activity compared across all treatments and controls. Fish treatments included Control (Con), High Temperature (HT), Low Temperature (LT), Re-use Water (RW), Salinity (Sal) and Handling/Confinement (Han). Values from all control tanks were pooled and presented as a single mean, which was compared to the mean for each stress treatment.

We measured plasma cortisol as an indicator of primary response and glucose, lactate, chloride and lysozyme as indicators of secondary stress responses to detect if stress responses occurred and to characterize differences in responses that are unique to each stressor or shared among stressors. Figures 1, 2 and 3 show the response of plasma glucose, lactate, chloride and cortisol concentrations in addition to lysozyme activity, respectively, compared across all treatments and controls. Values from all control tanks were pooled and presented as a single mean, which was compared to the mean for each stress treatment. Plasma glucose concentrations were significantly lower than control in high temperature and re-use water treatments, and significantly higher than control in the low-temperature treatment. Plasma lactate concentrations increased greater than 2-fold in high-temperature and saline treatments, and also increased after the handling treatment. In contrast, low temperature and re-use water treatments decreased plasma lactate concentrations. High temperature and saline treatments increased, while low temperature and re-use water treatments decreased plasma chloride concentrations compared to control. Lysozyme activity was slightly decreased in the re-use water treatment. Finally, plasma cortisol concentrations increased dramatically, between 5- to 12-fold, compared to the controls, across all treatment groups. The magnitudes and directions of the differences of these parameters between treatments and the control groups reflect unique physiological response portfolios that result from each stressor, such that a sequencing a collection of transcripts isolated from tissues from stressed fish would serve as a qualitative representation of various physiological responses.

### Analysis of gene expression

Stress and its resulting increase in cortisol levels have been reported to affect the onset of the apoptosis mechanism [50-52]. The expression of several apoptosis and stress-related candidate genes were analyzed in gill tissue using quantitative real-time PCR (qPCR) to determine if changes in expression of relevant genes occurs during the stress response.

**Figure 4:**
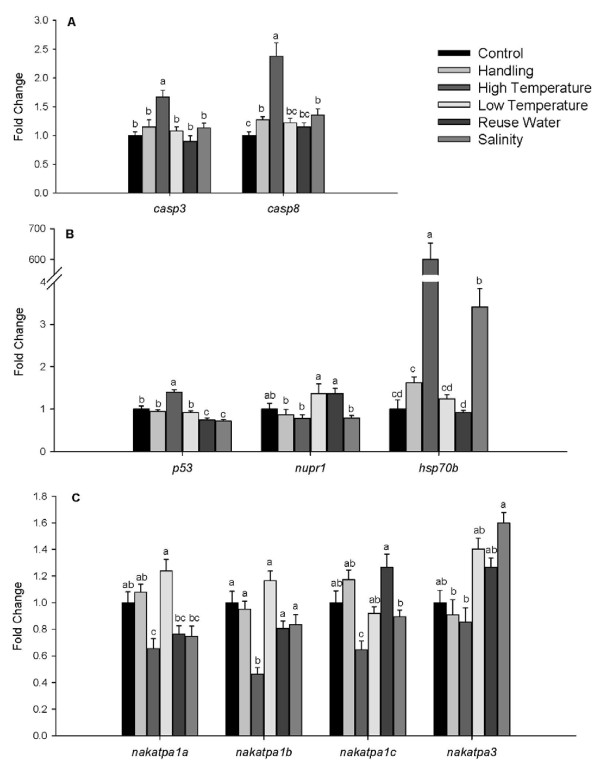
**Differential Expression in Response to Stress**. Figures 4a-c show the fold change in expression for genes involved in the stress response in gill tissues of fish within each treatment. Values represent fold change in expression compared to the control treatment, which was set at 1.0. Means without a common letter represent significant differences, P ≤ 0.05.

The caspases are proteolytic enzymes which are the core components of the intracellular apoptosis mechanism. Caspases often function in cascades, where an upstream caspase is activated by its interaction with a caspase adaptor [56,57]. Caspase 8 (*casp8*) is one of the upstream initiators known to trigger apoptosis, while caspase 3 (*casp3*) is activated downstream. Figure 4a represents the differential expression (in fold change) of *casp3 *and *casp8 *detected with qPCR. Compared to control, the expression of *casp3 *was up-regulated in the high temperature treatment only. *Expression of casp8 *increased in the handling, high-temperature, and salinity treatments.

QPCR results for tumor suppressor gene p53 (*p53)*, nuclear protein 1 (*nupr1)*, and heat shock protein 70 b (*hsp70b) *are reported in Figure 4b. *p53 *[58] has multiple functions, and can be induced by several stressors through different translation mechanisms; its major functions are to regulate apoptosis and growth arrest [59]. The expression of *p53 *was reduced in the reuse water and salinity treatments and increased in the high temperature treatment (Figure 4b). Nupr1 is also a multifunctional protein and similar to p53, this protein can be induced by different stressors and takes part in apoptosis regulation [60,61]. Momoda *et al. *[35] studied gene expression in rainbow trout liver after handling stress, and documented an increased expression after 3 hours of stress and even higher after 21 h. However, in the current study, *nupr1 *expression was not significantly different from the control for any of the stress treatments. Heat shock proteins (HSP) were initially identified as being induced after stressful thermal conditions; however, it is presently known that this group of proteins also responds to other environmental stressors [37,62]. Expression of h*sp70b *was up-regulated in high temperature and salinity treatments, with a dramatic 600-fold increase in expression with the high-temperature stress.

To compensate for osmotic gain of fluid and diffusive loss of ions, freshwater teleosts developed an active ion transport mechanism. Ions like Na^+^, Cl^-^, Ca^2+ ^are actively transported across the gill epithelium to maintain high levels of ions in the blood. Euryhaline fishes that move from freshwater to salt water transform their gills from ion absorbing mechanisms to an ion-secreting epithelium, resulting in an increase of Na+/K+-ATPase (*nakatp*) gene expression [63,64]. A previous study of *nakatp *expression in rainbow trout gill [64] found that isoforms *nakatpα1c *and *nakatpα3 *did not respond to salinity exposure, while isoform *nakatpα1a *decreased expression within one day after being transferred to salt water and isoform *nakatpα1b *gradually increased its expression. In this study, expression of the *nakatpα1a, nakatp*α*1b*, and *nakatpα1c *isoforms were significantly reduced by high temperature treatment only. Isoform *nakatpα3 *showed increased expression in fish exposed to the salinity treatment (Figure 4).

### Transcriptome sequencing and assembly

Newbler and MIRA3 were used to assemble 3,160,306 high quality reads generated by 454 pyrosequencing of the rainbow trout RNA samples; this information is available through the Short Read Archive in GenBank as Accession SRX085156. Detailed information on the assembly results is presented in Table 1. Newbler aligned 2,708,437 reads (85.7%) including 990,882,504 base pairs to contigs and 235,486 (7.4%) reads were identified as singletons. The sequences were assembled into 110,031 contigs with an average depth of 17.9× coverage; 90,417 of them were at least 100 base pairs long. The contigs were linked to form 83,166 isotigs in 41,879 isogroups. In addition, 1,123 contigs were entered in the assembly results of isotigs. Those contigs were larger than 500 base pairs and did not belong to any isotigs. Thus, the total number of sequences assembled into isotigs was 84,289. Additionally, 235,486 reads that presented a certain level of redundancy were considered singletons by Newbler. Those sequences were re-assembled using MIRA3. Consequently, 91,926 more reads were assembled, resulting in 37,143 MIRA3 contigs. This step improved the prediction of different gene variants. For example, there are two known variants of the apaf1 gene, and Newbler assembly only predicted one of them, but MIRA3 identified the other variant. Sequence and assembly information for contigs, isotigs, and isogroups are available at http://www.ars.usda.gov/Research/docs.htm?docid=8033.

**Table 1:** Detailed Information on the Assembly Results.

Newbler Reads	
Total number of reads	3,160,306
Number of aligned reads	2,708,437
Number of aligned bases	990,882,504
Number of wholly assembled reads	2,326,354
Number of partially assembled reads	374,202
Number of singletons	235,486
Number of repetitive reads	12,291
Number of outlier reads	139,210
Number of too-short reads	72,763
**Newbler Contigs**	

Total number of contigs	110,031
Total number of contig bases	55,246,326
Average coverage	17.9×
Average contig size	502 bp
Contig N50	829 bp
Number of contigs with size > 100 bp	90,417
Number of contigs with size > 500 bp	42,767
Largest contig Size	16,649 bp
**Newbler Isotigs**	

Total number of isotigs	83,166
Average contig count	3.7
Largest contig count	29
Number of isotigs with one contig	28,111
Average isotig size	1,337 bp
Isotig N50	1,639 bp
Largest isotig Size	24,959 bp
**Newbler Isogroups**	

Total number of isogroups	41,879
Average isotig count	2.0
Average contig count	2.6
Largest isotig count	649
Largest contig count	2,491
Number of isogroups with one isotig	28,615
Number of isogroups with one contig	27,530
**MIRA3 assembly of Newbler singletons**	

Reads assembled	91,926
Number of contigs	37,143
Contig N50	486
Largest contig size	1604

### Transcriptome characterization

For the Newbler assembly, only isotigs that are longer than 100 bp were selected for annotation. To avoid redundancy, all isotigs with 90% or higher identities were grouped in one cluster and only a representative from each group was selected for annotation. This step reduced the number of sequences from Newbler assembly from 83,935 to 62,071. Moreover, the 143,560 reads listed on MIRA3 results as debris (singletons), were masked with RepeatMasker using the INRA (National Institute for Agricultural Research, France) Rainbow Trout repeat and cGRASP Salmon masker databases, and 39,210 masked sequences that have more than 300 base pairs of continuous non repetitive segments were also included in the annotation process. After adding the 37,143 MIRA3 contigs; 138,424 sequences were included used for annotations.

**Figure 5:**
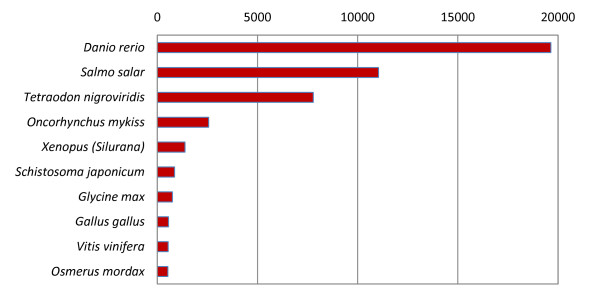
**Top Blast Hits by Species**. Figure 5 shows the species represented in the top BLASTx hit for each isotig.

Overall, 56,991 sequences were detected as BLASTx hits. The top 10 distributions of BLASTx species are shown in Figure 5, in which Zebrafish (*Danio rerio*) is the most counted species, followed by Atlantic Salmon (*Salmo salar*), Pufferfish (*Tetraodon nigroviridis*), and Rainbow Trout (*Oncorhynchus mykiss*). In the mapping step, GO annotation data for 50,991 sequences were retrieved from the GO Database. Default settings for Blast2Go annotation was used in the final annotation step and 43,382 sequences were annotated, among them, 29,856 are from Newbler isotigs/contigs, 5,608 are from MIRA3 contigs, and 7,918 are from the masked reads.

The GO term distribution (see Additional File 2 Figure S1) for the annotated sequences was distributed between level 2 and 13. For the molecular function group (F), the distribution peaked at level 3, the cellular component (C) and biological process (P) at level 6. GO annotation results of the sequences are presented in Additional File 1 (Table S2 Gene Ontology Annotation). In summary, of the 43,382 sequences that were assigned GO terms by B2G; 35,538 are involved in biological processes, 37,837 have molecular functions, and 36,316 are cellular components. Most of these sequences (86%) are distributed among two or three general GO term domains. In the biological process distribution, cellular process is the most abundant group (19%), followed by metabolic process (14%), and biological regulation (11%). Binding (51%), catalytic activity (26%), and molecular transducer activity (6%) are the top three distributed molecular functions. For cellular component, cell (44%) is the most found component, and other abundant cellular components are organelle (29%), macromolecular complex (12%), and membrane-enclosed lumen (9%). These results are presented in Additional File 2 Figure S2. To evaluate the representation of genes associated with stress response in the stress reference transcriptome, we searched the Hit Description and Gene Ontology Term annotation in Additional File 1 (Table S2 Gene Ontology Annotation) for keywords associated with the treatments in our study. Results in Table 2 show that a significant number of sequences were identified from annotation associated with stress responses.

**Table 2:** Keyword Search of Functional Annotation.

Search Term	BLASTx Hit Description	Gene Ontology Term
chloride	903	524
salinity	0	33
salt	49	127
water	0	200
oxygen	1210	827
carbon dioxide	0	8
ammonia	26	44
nitrate	19	10
lactate	90	34
cortisol	0	11
glucose	1221	1052
lysozyme	49	15
stress	304	1465
heat shock	759	191
temperature	31	67
apoptotic	89	157
apoptosis	821	3698

Searching BLASTx Hit Descriptions and Gene Ontology Terms associated with isotigs from the reference transcriptome reveals significant representation of transcripts expected to be involved in stress responses.

Current rainbow trout transcriptome sequence datasets available in NCBI include 287,967 EST sequences in dbEST and the 454-pyrosequencing data submitted to SRA, which include 1,298,911 sequences of a Swanson double-haploid fish and 1,507,274 other rainbow trout cDNA sequences submitted by INRA. In addition, there are 116,463 sequence clusters generated from 391,356 rainbow trout mRNA and EST sequences in NCBI UniGene database and 161,818 rainbow trout transcriptome reference sequences generated by Salem *et al. *[31]. We compared our sequence reads with all of these datasets, and found that 69.1%, 78.5%, and 56.2% of the reads can be mapped to the rainbow trout sequences in NCBI EST, NCBI SRA for double-haploid fish, and NCBI SRA from INRA, respectively. Moreover, only 67.5% and 55.2% of the reads can be aligned or partially aligned to the rainbow trout cluster sequences in NCBI UniGene database, and the transcriptome reference sequences of Salem *et.al*., respectively. These comparisons suggest that significant amounts of new data have been generated in our dataset.

### SNP Discovery

Four full sib families were used in this experiment, therefore assembly of the transcriptome data was expected to result in the identification of putative SNPs. A total of 57,207 SNPs were identified from the Newbler assembly which represents 30,293 contigs assigned to 21,335 isogroups. The average number of putative SNPs in a contig was 1.9 and in an isogroup 2.7. The maximum number of putative SNPs in a contig was 20, and 15,667 contigs had only one putative SNP. On average, one putative SNP was found every 483 bp. The maximum number of putative SNPs in an isogroup was 392, and 13,254 isogroups had at least two SNPs. Similarly, an additional 808 putative SNPs were identified from the MIRA3 assembly. Overall, the putative SNPs would include 37,358 transitions and 20,657 transversions, similar to the 2:1 ratio observed in some but not all species [65]. To determine the potential for sequence variation to influence gene function we identified the location of putative SNPs in relation to gene sequences based on BLASTx alignments. Overall, 24,479 putative SNPs were determined to fall within exons, 4907 of which were located in the first position of a codon and 4110 the second, both sets expected to impact amino acid sequence and potentially gene function. We also predicted that 15,321 putative SNPs are located in the third position, which as expected is much more than the first and second positions combined. An additional 1322 SNPs were determined to be within exons however discrepancies of SNP positions across multiple alignments resulted in uncertainty of codon position.

It is likely that some of the putative SNPs are actually paralogous sequence variants (PSVs), and therefore are the result of gene duplication events and not sequence variation [66]. In salmonids we expect a high frequency of PSVs due to an evolutionarily recent genome duplication event [67]. However, as all the sequences are transcribed differences may still be relevant to gene function as evolutionarily diverged paralogs sometimes have unique expression profiles [68]. These putative SNPs will be incorporated into a data set previously isolated from a genomic DNA reduced representation library [69] for genetic analyses in rainbow trout (Additional File 3 Tables S3 and S4). Although it would be interesting to evaluate these SNPs for allelic specific expression, our combining multiple tissues from multiple individuals for the creation of a normalized library for this experiment would not produce meaningful quantitative data. However, additional studies of these samples using RNA-Seq will permit such an analysis, yet we must first determine how to distinguish differential expression of allelic variants from that of closely related genes resulting from the genome duplication events.

## Conclusions

We have generated and characterized a reference transcriptome for rainbow trout that represents multiple tissues responding to multiple stressors, and the unique expression portfolios for each. This resource complements existing public transcriptome data and will serve approaches aiming to evaluate gene expression associated with stress in this species.

## Methods

All experiments were conducted under approval of the USDA/ARS National Center for Cool and Cold Water Aquaculture Institutional Animal Care and Use Committee, protocol #50.

### Fish

Four unrelated families from the NCCCWA even year broodstock population under selective breeding for growth [70] were used for each of the five stressors and control treatments. The fish were tagged and two fish from each family were placed in each of four 193 liter tanks on flow-through water. For each stressor the four tanks included three replicates and one control; a total of 160 fish were used. The experimental fish were acclimated during a two week period at NCCCWA standard culture conditions (13.7°C; CO_2 _25.0 mg/l; DO 15.1 mg/l; salinity 0 ‰). The fish were weighed at the end of the trial, and had average weight of 57.7 g (range: 28.0-99.0 g). Water quality parameters (DO, CO_2_, temperature and nitrogenous compounds) were monitored during the acclimation period and at the beginning (time 0), middle (time 3) and end (time 6) of the stress treatments.

### Stress challenges

After acclimation, water conditions in each tank were brought to the experimental parameters over the course of three hours (time 3) and then maintained at stress conditions for three hours. The following stress conditions were used: 1) high temperature 25°C; 2) low temperature 2°C; 3) salinity 32‰ (addition of salt, NaCl); 4) Re-use water, presenting lower DO and higher CO_2_; and finally 5) handling/crowding stressors, following procedures previously described by Weber and colleagues [71] as adapted from Pottinger *et al. *[44]. Following each stress treatment, all eight fish from each tank were euthanized by administering a lethal dose of MS-222 and tissues (blood; brain; gill; heart; kidney; liver; spleen and muscle) were collected immediately and frozen in liquid nitrogen.

### Plasma metabolite measurements

To identify the physiological responses to stress, plasma metabolite measurements including concentrations of plasma cortisol, glucose, lactate, and chloride in addition to lysozyme activity were measured. Whereas cortisol is a measure of the primary stress response, the others are measures of secondary stress responses. Glucose and lactate serve as measures of metabolic response, chloride as a measure of the osmoregulatory response, and lysozyme activity as a more direct effect of the stressor on the immune system.

Plasma cortisol was measured by radioimmunoassay following procedures described by Redding *et al. *[72], as modified by Feist *et al. *[73], but using cortisol antiserum R4866 (provided by Coralie Monroe, University of California-Davis School of Veterinary Medicine, Department of Reproduction), which was validated for use in rainbow trout (Barry *et al. *[74]). Plasma samples were exposed to heat to denature binding proteins. Glucose was measured by enzymatic coupling with hexokinase and glucose 6-phosphate dehydrogenase (Infinity glucose [HK] liquid stable reagent, Thermo Trace, Melbourne, Australia). Lactate was measured by enzymatic conversion of lactate plus NAD to pyruvate plus NADH (lactate dehydrogenase, Sigma, St Louis MO, #L-2500; NAD Sigma #N-7004). Chloride was measured using a Labconco model 4425000 digital chloridometer (Kansas City MO). Plasma lysozyme activity was measured using a lysozyme turbidity assay as described by Muona and Soivio [75] as modified by Vivas *et al. *[76]. The bacterium used was *Micrococcus lysodeikticus *(Sigma, St Louis MO, M3770) and lyophilized hen egg white lysozyme was used as standard (Sigma, L-6876).

### Quantitative PCR

Differential expression among the stressors was determined by sampling gills from all the stress and control samples (160 samples) for quantitative PCR (qPCR). RNA was isolated from tissue using standard TRIzol (Invitrogen, Carlsbad, CA) extraction methodology according to the manufacturer's suggested protocol. RNA quality was visualized using agarose gel electrophoresis. RNA samples were from those extracted for creation of the pooled library, see below. Reactions optimized for the following pathways and genes: 1) apoptosis pathway (*casp3; casp*8; *nupr1 *and *p53*); 2) Heat-shock protein (*hsp70b*); 3) *Na/KATPases *(*nakatpα1a; nakatpα1b; nakatpα1c *and *nakatpα3*). PCR reactions were run in duplicate for each sample using cDNA generated from DNase (Progema, Madison, WI)-treated RNA and a single reverse transcription reaction that included oligo-dT primers (Promega) and Moloney murine leukemia virus reverse transcriptase (Promega). DNase- and RNase-free water (Invitrogen) was used for all dilutions and reverse transcriptase and PCR reactions. Primers were designed using primer3 software. Primer sequences used for PCR reactions are provided in Table 3. All primers were optimized using a standard curve approach until PCR efficiency values fell between 1.85-2.15. All primers produced a single amplicon, which was confirmed as a single band on an agarose gel and a single peak from a melt-curve analysis.

**Table 3:** Quantitative PCR Gene and Primer Information.

Gene	Gene Symbol	Primer Sequences	Amplicon Size (bp)
Caspase 3	*casp3*	TTTGGGAGTAGATTGCAGGG TGCACATCCACGATTTGATT	52
Caspase 8	*casp8*	CAGCATAGAGAAGCAAGGGG TGACTGAGGGGAGCTGAGTT	93
P53	*p53*	GTGGAATTTGATCCGAGTCTGT AGTGTCCAGGGTAGAAATGGAG	78
Nuclear protein 1	*nupr1*	CGAAGAAGCACACTACGATCAA TCAGTCCGATTTCTCTCTTGGT	98
Heat shock			167
protein 70 b	*hsp70b*	AGGCCCAACCATTGAAGAGA GCAATGTCCAGCAATGCAATA	
NaKATPalpha1a	*nakatpa1a*	GTGACGGTGAGAAGAAGAACATC GGCAGAGACGATACGCAAAT	107
NaKATPalpha1b	*nakatpa1b*	GTCTTCCTGGGCGTGTCTTT CTCTGGCACATTAGCAACGAT	105
NaKATPalpha1c	*nakatpa1c*	ATCGTGACTGGTGTTGAAGAAG GAAGAAGAGGAAGGGTGTGATTTC	108
NaKATPalpha3	*nakatpa3*	GGCATCACCTTCTTCATCCT ACAGTAGCCAGTAAGCCCTCAG	113
β-actin	*Bact*	GCCGGCCGCGACCTCACAGACTAC CGGCCGTGGTGGTGAAGCTGTAAC	73
Elongation factor			95
1α	*1f1a*	CATTGACAAGAGAACCATTGA CCTTCAGCTTGTCCAGCAC	

Housekeeping genes were used to normalize the transcript expression values [Elongation factor (*1f1-α*) and *β-*actin (*bact*) [77,78]]. The geNorm applet for Microsoft Excel [72] was used to test the gene expression stability for the candidate genes (M = 0.595 for both *1f1α *and *bact*), and their geometric mean was used to generate a normalization factor for each sample. The normalization factor was used to calculate the expression of each gene relative to the geometric mean expression of the selected reference genes. Expression values were calculated as a fold change, relative to the expression of control group, which was set at 1.0. Fold change data were normalized using log_2 _transformation prior to statistical analysis.

Significant effects of stress on gene expression were detected using the general linear models procedure using PC-SAS (Version 9.1). In the event of a significant p-value (P ≤ 0.05), differences between treatments were detected using the Fischer's least significant difference (LSD) procedure. Data on metabolites were analyzed using SigmaStat (Version 3.1; Jandel Scientific, San Rafael, CA). Significant effects of stress on metabolite expression were detected by comparing among control and all treatment groups by Kruskal-Wallis One Way Analysis of Variance on Ranks, followed by Dunn's Method for all pairwise multiple comparisons of means. Treatment means were considered significantly different when P ≤ 0.05. All data are presented as means ± SEM (standard error of the mean).

### Transcriptome sequencing

To identify the collection of genes expressed during response to stressors, RNA was extracted from gill, brain, liver, spleen, kidney and muscle from one random sample from each treatment tank plus three control samples (total of 108 RNA samples). All RNA samples were diluted in water and pooled together in a 500 ng/μl concentration. Library preparation and sequencing were performed at the Indiana University's Center for Genomics and Bioinformatics. Methodology was based on Meyer *et al. *[23], with modifications by K. Mockaitis as described by Schwartz *et al. *[22]. Briefly, cDNA was synthesized from the RNA pool using primers optimized for GS FLX Titanium sequencing and dsDNA was generated by PCR amplification. Then, cDNA was normalized to reduce sequence coverage of highly expressed transcripts. Accordingly, hybridization and double-stranded nuclease (DSN) digestion were used for the normalization. To optimize sequencing efficiency, libraries were titrated by emulsion PCR/bead enrichment, performed as suggested by Roche 454. Transcriptome sequencing was performed using GS FLX Titanium (Roche 454) pyrosequencing technology.

### Transcriptome sequence assembly

The generated rainbow trout cDNA sequence reads were quality trimmed and the adaptor's sequences were removed. Subsequently, GS *De Novo *Assembler (Newbler) v 2.5p1 was used for sequence assembly. Default parameters for the algorithm of transcriptome assembly were selected, except for specific threshold parameters for generating isotigs. To further ensure the quality of the input reads, the contaminant database included in SeqTrim [79] package for screening of contaminant sequences such as those of *Escherichia coli *and cloning vectors was used as the optional trimming database of Newbler, in which a read is screened out if it entirely matches with a sequence in the contaminant database. The transcriptome algorithm of Newbler aligns one read completely or partially to a contig. This pipeline also identifies whether a read is repetitive, a singleton or too short (< 50 base pairs) to be used in the computation, or even if it is problematic for assembly. Portions of repetitive reads were included in the assembly results if they aligned to unique contigs. A unique feature of Newbler v2.3 and higher, is the ability of inferring splice-variants in its transcriptome assembly algorithm, in which all contigs are grouped into collections called isogroups based on the contig branching structures and the contig branching structures in each isogroup are further traversed, resulting in a set of isotigs, each representing a possible traversing path of the contigs in the isogroup. Therefore, there is a hierarchical structure among isogroups, isotigs, and contigs. An isogroup, which is analogous to a gene, is composed of a set of isotigs, which can be thought as all possible isoforms (splicing variants) of the gene; and an isotig contains one or more contigs, which can be roughly considered as exons in the gene as demonstrated by Ewen-Campen *et al. *[80]. In our study, some threshold parameters for generating isotigs were set to the maximum allowed values: the maximum number of isotigs in an isogroup was set to 1000, the maximum number of contigs in an isogroup to 1000, and the maximum number of contigs in one isotig to 200. These parameters enabled the program to generate all possible isotigs for most isogroups. After analysis, several reads were considered singletons by Newbler which included similar sequences, therefore Newbler singleton sequences were re-assembled using MIRA3 (MIRA-3.2.1).

### SNP discovery

For SNP identification, high quality sequence reads were first screened for repetitive elements with RepeatMasker [81], using the cGRASP Salmon repeat library [82] and the INRA Rainbow Trout repeat library [83] as reference. Using ssaha2 [84], repeat masked sequences with less than 100 base pairs of consecutive unmasked bases were mapped to the assembly contigs that were longer than 100 base pairs. Then ssaha_pileup pipeline was used to detect sequence variations among the mapped sequences. In order to increase the accuracy of the prediction, the identified putative SNPs were then further processed. First, putative SNPs having total component reads less than five or the component reads for the minor allele less than two were excluded. Then, to address the homopolymer errors commonly found in 454 sequences, only putative SNPs that were at least 50 base pairs apart from other putative SNPs were included. Additionally, flanking sequences were required to be at least 25 base pairs long. Alignment results for the Newbler isotigs and MIRA3 contigs from the BLASTx phase of the BLAST2GO annotation (see below) process were queried to identify query start, query end, and SNP position. These data were used to identify whether or not the SNP occurred inside an exon; if so they were used to calculate if the SNP is in the first, second or third position of the codon. Only data that was in agreement across all BLASTx alignments for each query was considered a positive result.

### Sequence Annotation

Functional annotations of Newbler isotigs/contigs, MIRA3 [85,86] contigs, and unassembled reads were performed using Blast2GO (B2G) [87], a Gene Ontology (GO) based sequence annotation tool. The Blast step was performed using Blastx search against the NCBI non-redundant (nr) protein database, with the expectation value threshold set to 1.0e^-6^. Isotigs were first processed with CH-HIT to remove redundant sequences before being used in the annotation step. All isotigs with 90% or higher identities were grouped in one cluster and only a representative from each group was picked for annotation. Functional annotations of Newbler isotigs/contigs and MIRA3 contigs were performed using Blast2GO.

### Sequence datasets comparison

A total of 3,091,890 sequences that had been cleaned of vector and *E. coli *sequences with SeqTrim were compared with the rainbow trout sequences in public available databases using SAHHA2 with the command line parameters: -454 -seeds 5 - score 60 -kmer 13 -skip 4 -diff 0 -output cigar -identity 95. The datasets compared are rainbow trout sequences in dbEST release 080111, sequences in NCBI SRA with the Accessions of SRX007396, SRX041526 - SRX041537, sequence clusters of NCBI UniGene Omy build 32, and the rainbow trout transcriptome reference sequences downloaded from NAGRP Aquaculture Genome Projects (http://www.genome.iastate.edu/aquaculture/salmonids/rainbowtrout/EST_WV.html).

## Authors' contributions

CER, JY and CCS were responsible for overall study design and execution, GMW was responsible for physiological data, BMC for quantitative PCR, GG performed sequence assembly and annotation. All authors have read and approved the final manuscript.

## Supplementary Material

Additional File 1 **Table S1 Water Quality Parameters, Table S2 Gene Ontology Annotation**. Table S1 contains temperature, dissolved oxygen, carbon dioxide, pH, ammonia, and nitrite data for each of the time periods and treatments. Table S2 contains transcriptome annotation assignments including BLASTx hits and Gene Ontology terms. Additional file 1: Additional File 1 Water Quality and GO.xlsx, 11694Khttp://www.biomedcentral.com/imedia/8161263926490264/supp1.xlsx. Click here for file

Additional File 2 **Gene Ontology Figures, Figures S1 and S2 in a PDF**. Figures show the distribution of Gene Ontology hits by GO level as broken down by Biological Brocess (P), Molecular Function (F) and Cellular Component (C) (Figure S1). A second figure shows assignment of Gene Ontology categories broken down by Biological Processes (a), Molecular Functions (b) and Cellular Components (c) (Figure S2). Additional file 2: Additional File 2 Gene Ontology Results.pdf, 934K http://www.biomedcentral.com/imedia/1534221861652145/supp2.pdf Click here for file

Additional File 3 **Table S3 Newbler SNP Discovery, Table S4 MIRA3 SNP Discovery**. Table S3 contains information for 57,207 putative SNPs identified from Newbler reference transcriptome contigs. Table S4 contains information for 808 putative SNPs identified from MIRA3 reference transcriptome contigs. Additional File 3: Additional File 3 SNPs.xlsx, 11083K http://www.biomedcentral.com/imedia/8523938676521459/supp3.xlsx Click here for file
