# Supplementary figures and images for: Generation of a reference transcriptome for evaluating rainbow trout responses to various stressors
Source: BMC Genomics. 2011 Dec 21;12:626. doi: 10.1186/1471-2164-12-626 (PMC3305546; doi:10.1186/1471-2164-12-626)

Figure S1 GO-level Distribution

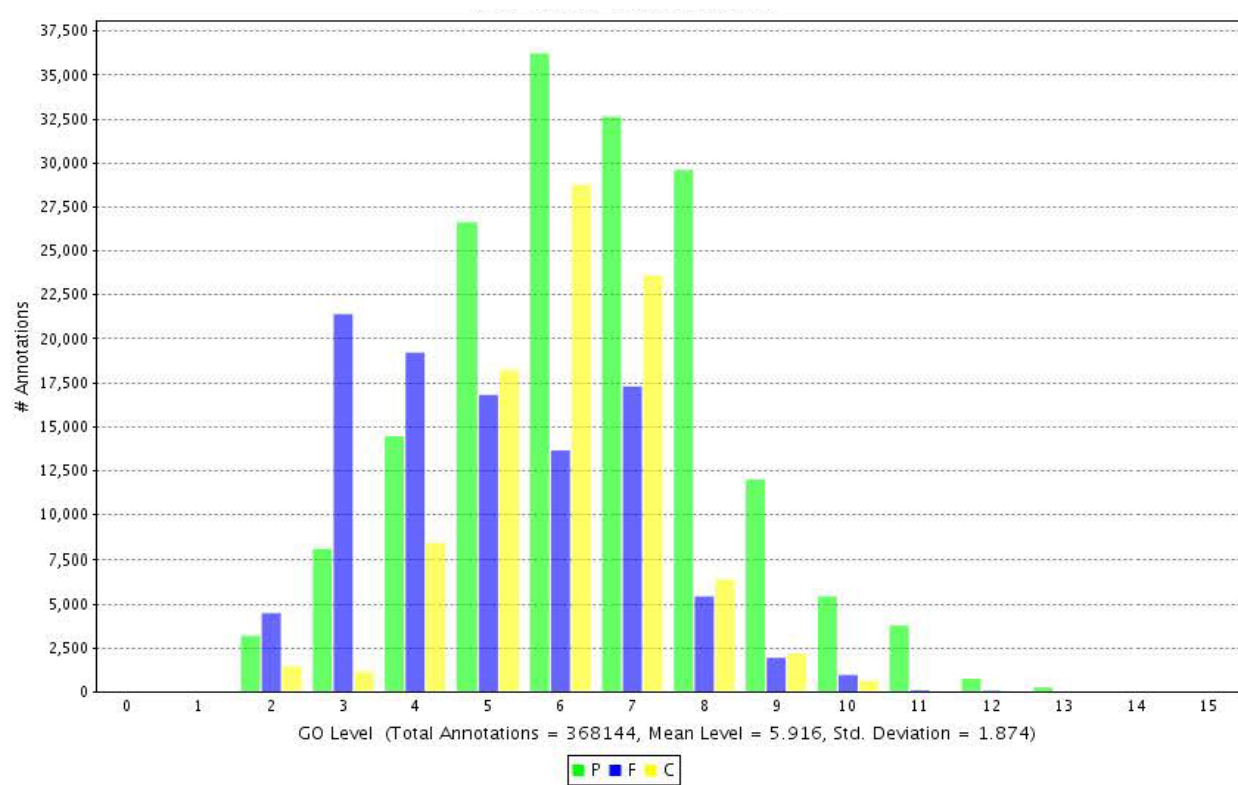

Figure S2 Breakdown of Gene Ontology Results

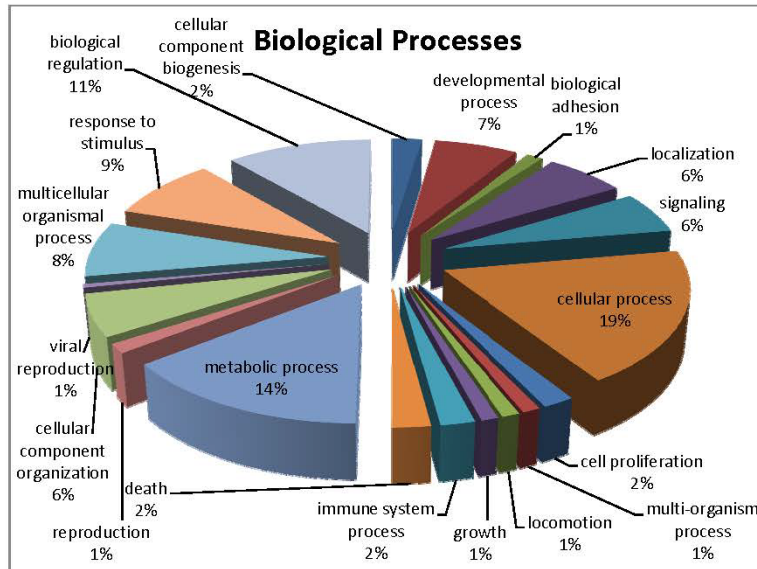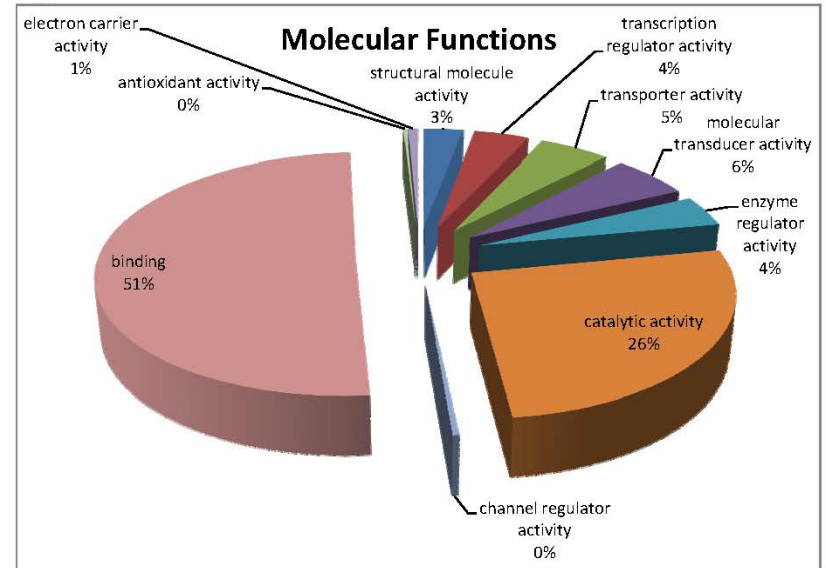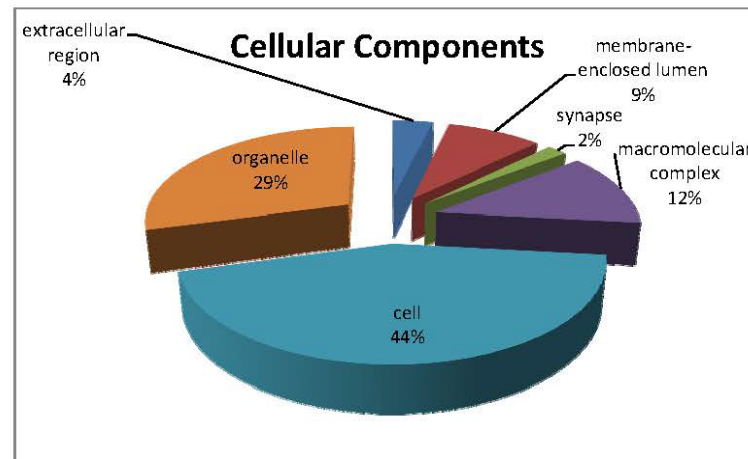

Supplement: Additional File 2 — Gene Ontology Figures, Figures S1 and S2 in a PDF. Figures show the distribution of Gene Ontology hits by GO level as broken down by Biological Brocess (P), Molecular Function (F) and Cellular Component (C) (Figure S1). A second figure shows assignment of Gene Ontology categories broken down by Biological Processes (a), Molecular Functions (b) and Cellular Components (c) (Figure S2). Additional file 2: Additional File 2 Gene Ontology Results.pdf, 934K http://www.biomedcentral.com/imedia/1534221861652145/supp2.pdf [file 1471-2164-12-626-S2.PDF]
